# Development of a decision guide to support the elderly in decision making about location of care: an iterative, user-centered design

**DOI:** 10.1186/s40900-016-0040-0

**Published:** 2016-07-19

**Authors:** Mirjam M. Garvelink, Julie Emond, Matthew Menear, Nathalie Brière, Adriana Freitas, Laura Boland, Maria Margarita Becerra Perez, Louisa Blair, Dawn Stacey, France Légaré

**Affiliations:** 1 CHU de Québec Research Centre - Hôpital St-Francois d’Assise, 10 Rue Espinay, Quebec City, QC G1L 3L5 Canada; 2 Centre de santé et de services sociaux de la Vieille-Capitale, 880, rue Père-Marquette, Quebec City, QC G1M 2R9 Canada; 3 Ottawa Hospital Research Institute, 725 Parkdale Ave., Ottawa, ON K1Y 4E9 Canada; 4 University of Ottawa, 451 Smyth Road, Ottawa, ON K1H 8M5 Canada; 5 Department of Family Medicine and Emergency Medicine, Faculty of Medicine, Université Laval, 1050, Ave de la Médecine, Pavillon Ferdinand-Vandry, Quebec City, QC G1V 0A6 Canada

**Keywords:** Decision aids, End-user involvement, Caregivers, Elderly, Location of care, Shared decision making

## Abstract

**Plain English summary:**

For the elderly to get the care and services they need, they may need to make the difficult decision about staying in their home or moving to another home. Many other people may be involved in their care too (friends, family and healthcare providers), and can support them in making the decision. We asked informal caregivers of elderly people to help us develop a decision guide to support them and their loved ones in making this decision. This guide will be used by health providers in home care who are trained to help people make decisions. The guide is in French and English. To design and test this decision guide we involved elderly people, their caregivers and health administrators. We first asked them what they needed for making the decision, and then designed a first version of the guide. Then we asked them to look at it and give feedback, which was used to make the final version. We then used scientific criteria to check its content and the language used. The final decision guide was acceptable to the caregivers, their elderly loved ones, and the health administrators. The guide is currently being evaluated in a large research project with home care teams in the province of Quebec.

**Abstract:**

**Background**

As they grow older, many elderly people are faced with the difficult and preference-sensitive decision about staying in their home or moving to a residence better adapted to their evolving care needs. We aimed to develop an English and French decision aid (DA) for elderly people facing this decision, and to involve end-users in all phases of the development process.

**Methods**

A three-cycle design with involvement of end-users in Quebec. End-users were elderly people (*n* = 4) caregivers of the elderly (*n* = 5), health administrators involved in home-care service delivery or policy (*n* = 6) and an interprofessional research team (*n* = 19). *Cycle 1*: Decisional needs assessment and development of the first prototype based on existing tools and input from end-users; overview of reviews examining the impact of location of care on elderly people’s health outcomes. *Cycle 2*: Usability testing with end-users, adaptation of prototype. *Cycle 3*: Refinement of the prototype with a linguist, graphic designer and end-users. The final prototype underwent readability testing and an International Patient Decision Aids (IPDAS) criteria compatibility assessment to verify minimal requirements for decision aids and was tested for usability by the elderly.

**Results**

*Cycle 1:* We used the Ottawa Personal Decision Guide to design a first prototype. As the overview of reviews did not find definitive evidence regarding optimal locations of care for elderly people, we were not able to add evidence-based advantages and disadvantages to the guide. *Cycle 2:* Overall, the caregivers and health administrators who evaluated the prototype (*n* = 10) were positive. In response to their suggestions, we deleted some elements (overview of pros, cons, and consequences of the options) that were necessary to qualify the tool as a DA and renamed it a “decision guide”. *Cycle 3:* We developed French and English versions of the guide, readable at a primary school level. The elderly judged the guide as acceptable.

**Conclusion**

We developed a decision guide to support elderly people and their caregivers in decision making about location of care. This paper is one of few to report on a fully collaborative approach to decision guide development that involves end-users at every stage (caregivers and health administrators early on, the frail elderly in the final stages). The guide is currently being evaluated in a cluster randomized trial.

**Trial registration:** NCT02244359.

## Background

The general population is aging [1, 2], and most elderly people sooner or later face the difficult decision about whether they should continue to live at home or move and receive care in another location [3]. This decision is preference-sensitive [3, 4], meaning it should be made with consideration of the elderly person’s preferences and values. Typically, multiple stakeholders are involved in the care of the elderly, including various healthcare professionals and informal caregivers (often family members or significant others) who should share the decision making process with the elderly person and/or their caregiver [5]. An interprofessional shared decision making (or IP-SDM) approach is the most appropriate process because it recognizes multiple stakeholder contributions to the decision-making process [6]. With an IP-SDM approach, all stakeholders consider the best available evidence regarding the risks and benefits of available options and make explicit their own values and preferences for particular options, but agree to focus on the decisional needs of the senior [7, 8].

Decision aids (DAs) are effective tools for promoting SDM [9], and could also be used to support IP-SDM. DAs are designed to guide people through the decision-making process by making explicit the decision, outlining options and associated consequences, and considering values and preferences relating to the decision [10]. DAs can be generic (i.e. useful for a variety of decisions) or tailored to specific decisions. A Cochrane review of 115 trials showed that DAs improve knowledge and congruence between a person’s values and the chosen option and decrease decisional conflict and passivity in decision making [10].

Within a longstanding research program aiming to promote IP-SDM approaches in healthcare [6–8, 11, 12], we recently initiated a multicenter cluster randomized trial evaluating the impact of a multifaceted intervention to increase SDM for location of care decisions involving the elderly and their interprofessional home-care teams in Quebec, Canada (DOLCE study) [4]. This research project included the development of a decision guide as a core component of our practice-based research on interventions that could help home care teams support decision making about location of care among the elderly. User-centered design is an approach that involves end-users in designing applications, and has been shown to increase the usability of applications [13, 14]. This project built on earlier successful partnerships with caregivers and health administrators whose needs and input have guided the entire project from conception to implementation. In this article we describe the user-centered design approach adopted to develop this guide.

## Methods

### Aim

Based on the needs and reactions of end-users (the elderly, their caregivers, and health administrators), we sought to develop a decision aid for the decision faced by elderly people, their informal caregivers and their health professionals about whether to continue to live at home or move into residential care.

### Design and development process

To structure the development process, we chose an iterative, user-centered design with three cycles in which we partnered with caregivers of frail elderly people, elderly people, and health administrators to understand their needs and goals and incorporate relevant changes in the decision aid (Cycles 1–3) [13, 14]. In brief, we developed (Cycle 1) and refined (Cycles 2–3) a series of prototypes of the guide, adjusting them according to end-user feedback [14]. After each substantive adjustment, team members met to discuss the changes and next steps (total of 10 meetings). The development process was also informed by the International Patient Decision Aids Standards (IPDAS) [15, 16], the literature [17–19], and other DAs for the elderly [20–22]. English and French versions of the guide were developed simultaneously to meet the language requirements of the target patient population.

The development of our guide was part of the DOLCE trial, for which ethical approval was obtained from all participating health and social services centers in the region of Quebec (CÉR du CSSS Vieille-Capitale/Québec-Nord/Portneuf; CÉR du CSSS de Rimouski-Neigette; CSSS de La Matapédia; CSSS de Lac-Saint-Jean-Est; CSSS de Kamouraska; CSSS de Maskinongé; CSSS de Beauce; CSSS de Montmagny-L’Islet; CSSS de Trois-Rivières; CSSS de Rocher-Percé; CSSS de Charlevoix; CSSS de Jonquière; CÉR du CSSS Alphonse-Desjardins; CÉR du CSSS de Chicoutimi; MP-CHU-QC-14-001).

### Minimal scientific criteria for DAs

Each prototype was assessed for compatibility with all 12 minimal IPDAS criteria: 1) qualifying criteria, required in order for an intervention to be considered a DA (six items); and 2) certification criteria, without which a DA is judged to have a high risk of harmful bias (six items) (Table 1) [16]. Guides to support decision-making that do not fulfil these six criteria are not DAs.

**Table 1:** IPDAS minimal qualifying and certification criteria for decision aids

			Prototype 1	Prototype 2	Final tool
Criterion					
Qualifying criteria	1	DA describes health condition or problem for which index decision is required	✓	✓	✓
	2	DA explicitly states the decision that needs to be considered (index decision)	✓	✓	✓
	3	DA describes the options available for the index decision	✓	✓	✓
	4	DA describes the positive features (benefits/advantages) of each option	✓		
	5	DA describes the negative features (harms, side effects, or disadvantages) of each option	✓		
	6	DA describes what it is like to experience the consequences of the options (physical, psychological, social)	✓		
Certification criteria	7	DA shows the negative and positive features of options in equal detail (using similar fonts, sequence, and representation of statistical information)	✓		
	8	DA (or associated documentation) provides citations to the evidence selected	✓	✓	✓
	9	DA (or associated documentation) provides a production or a publication date	✓	✓	✓
	10	DA (or associated documentation) provides information about the update policy	✓	✓	✓
	11	DA provides information about the levels of uncertainty around event or outcome probabilities	✓	✓	✓
	12	DA (or associated documentation) provides information about the funding source used for development	✓	✓	✓

*DA* decision aid

### Participants

The guide was developed by the DOLCE study team in collaboration with caregivers of elderly people who had been previously involved with location of care decisions for their loved ones (*n* = 5) and health administrators (*n* = 6) involved in home-care service delivery or policy implementation in Quebec, who would provide the guide for their clients. Elderly people evaluated a final version of the guide.

The research team (including advisory board) comprised members in the fields of family medicine (FL), nursing (DS), (home care) management or policy (NB, JE, NT, DG, JG, TS, ST, FB, FF, CA, SGLM), SDM (MG, FL, DS, LB), public health (MM, MMBP), population health (LB), and two caregiver representatives (HB, LR). Together, the team has expertise in DAs and knowledge of the home-care context.

Three additional caregivers (for a total of five) were invited to become partners in the research and acted as mentors to DOLCE study trainees (MG, JE, MM, LB, MMBP) who met with their mentors on a regular basis. In these meetings the mentors also received information about the DOLCE project (which included the development of this guide), discussed their role in the project and what would be expected of them, and signed an informed consent form to this end, as required by our funding organization.

Six health administrators acted as advisors to this study, including one representative from the Quebec Ministry of Health and Social Services, three directors of health and social services centers, and two directors of regional health agencies in Quebec. These experts were involved even before we applied for a grant to conduct the project (as well as for ethics approval), and gave their verbal informed consent to participate as advisory board members in the project and on the grant application.

The third category of end-users were the elderly themselves. However, we thought the elderly patients would find it burdensome and confusing to engage in the arduous, fastidious and repetitive iterative process of tool development. Elderly people being cared for by our caregiver end-users were therefore consulted for usability testing only in the final stages of development.

Caregivers rather than the elderly people in their care were the principal end-users consulted in the earlier stages of developing the guide for several reasons: a) preliminary data from the DOLCE study indicates that the majority of people confronted with this type of decision are caregivers of cognitively impaired elderly people; b) they are the ones who experience the most decisional conflict during the decision-making process [23, 24] and yet have hitherto been largely ignored in research about elderly people [24]; c) in general, caregivers have shown themselves to be most critical of the care their loved ones received, while elderly people themselves tend to be more accepting; d) the majority of caregivers, fairly elderly themselves (over 65 years old), will be having to decide whether to move themselves in the not-so-distant future.

### Cycle 1 (March 2013–October 2014)

#### Needs assessment

We identified decisional needs regarding elderly people’s location of care. First, as part of an earlier study, we interviewed six caregivers of elderly people who had recently faced a decision about location of care. Detailed information about this study can be found elsewhere [7]. Second, research team members met with caregivers (*n* = 5) to understand their experiences of decision making for the senior, their needs and goals related to location of care decisions, and to receive input on prototypes of the guide.

#### Search for existing DAs

We searched the Ottawa Hospital Research Institute DA inventory (https://decisionaid.ohri.ca/AZinvent.php) for existing DAs about location of care and for generic DAs. This international inventory contains all publicly available DAs on a variety of health topics.

#### Evidence about the options

To populate the guide with benefits and risks about location of care options, the research team members conducted an overview of reviews [25] (Boland et al., submitted 2016). With help from an information specialist we searched MEDLINE, the Cochrane Library, EMBASE and CINAHL. Additionally, we contacted authors of a relevant Cochrane review that was being updated [26]. We included reviews that evaluated health and wellbeing outcomes related to location of care for people aged 65+ years. All languages were eligible. Quality of the reviews was assessed with the AMSTAR checklist. A sensitivity analysis was conducted with higher quality systematic reviews. Detailed results of this pilot umbrella review can be found elsewhere (Boland et al., submitted 2016).

#### Drafting a first prototype

We drafted a first prototype, integrating information from the pilot umbrella review into an existing guide. We adjusted the presentation of information to the target population (e.g. font size, lay-out) and considered user literacy (e.g. minimized use of multi-syllable words, number of words per sentence).

### Cycle 2 (October 2014–January 2015)

#### Usability testing I

To assess guide usability, we approached the same five caregivers and five of the six health administrators involved, to complete a survey (Table 2: Caregivers). Quantitative data was imported into an Excel file and analyzed descriptively. For continuous data, we calculated the median and range. We summarized open-ended comments by topic and end-user type (caregiver vs. health administrator). All findings were discussed by our research team until consensus was reached on how to integrate them into the guide.

**Table 2:** Usability test

Caregivers (answer categories “0-not at all”, “1-somewhat”, “2-fairly”, “3-very much”) • Is the language in the decision aid understandable? • Are you satisfied with the length of the decision aid (8 pages)? • Is the presentation of the decision aid right for its target group and purpose (lay-out, size, font size, use of pictures)? • Does the decision aid provide you with enough information? (if not, please indicate what information is missing in “ open comments”) • Is it clear how the decision aid should be used (the steps to take)? • Do you think the decision aid would be helpful for seniors and their caregivers who are facing a decision about location of care? • I found the presentation: Slanted towards staying at home/Balanced/Slanted towards moving elsewhere *(this question was added later on)*. • Other comments (open) Elderly • Please rate each section of the decision aid, by circling “4-Everything clear”, “3-most things clear”, “2-some things unclear”, “1-many things unclear” to show what you think about the clarity of the information • The length of the decision aid was: Too short/Just right/Too long • The amount of information was: Too little /Just right /Too much • I found the presentation: Slanted towards staying at home/Balanced/Slanted towards moving elsewhere • Do you think the decision aid would be helpful for people making this decision? Yes / No • Do you think the decision aid would be acceptable to use with people making this decision? Yes / No	

#### Adjusting the prototype

The guide was continuously adjusted according to ongoing feedback from end-users, who met with the research team members on regular basis, and team members’ reflections.

### Cycle 3 (February 2015–May 2015)

#### Refining the prototype (editing texts, lay-out)

French and English linguistic experts reviewed all final content to enhance understandability. A graphic designer developed the layout of the guide.

#### Readability

We assessed whether the final guide was readable at a Grade 5 level (uncompleted primary school) with readability software. For the French version, we used “Scolarius”, a combined measure of multiple readability tests that ranges from 50 to 190+. We aimed for scores between 50 and 89. For the English version we used “Readability Plus”, which uses the Flesch Reading Ease scale of 0-100, with a higher score indicating easier readability. We aimed for scores of 60 + .

#### Usability testing II

Elderly people assessed the final guide for its usability, completing similar questionnaires as did caregivers and health administrators in Cycle 2 (see Table 2: Elderly). We approached the elderly family members of the caregivers involved in this project, as well as some participants in the DOLCE study intervention group.

## Results

### Cycle 1

#### Needs assessment

The interviews, conducted as part of a previous project, indicated that caregivers did not perceive that a SDM process was occurring between them, the senior and the home care team when deciding about whether or not to relocate care for their loved one and did not always feel supported in decision-making, highlighting a need for effective interventions to implement IP-SDM in home-care contexts [7]. Five meetings with caregivers as part of the current project shed further light on their experiences with the decision-making process, and how a guide could be helpful.

#### Search for existing DAs

We found six existing DAs about location of care for the elderly [20–22, 27]. However, these were excluded as they targeted specific populations (e.g., veterans, elderly people with dementia, caregivers only), or did not fulfill all IPDAS minimal criteria. We also identified the Ottawa Personal Decision Guide (OPDG), a generic decision guide based on the Ottawa Decision Support Framework [28] that when populated with decision specific information on options, benefits and harms, fulfills the minimum quality criteria for DAs [29]. The OPDG, which has proven supportive in decision making [30, 31], is a two-page guide with four steps: 1) clarify decision; 2) explore decision; 3) identify decision making needs; 4) plan next steps based on needs. We used the OPDG as the basis of our guide. The generic version of the OPDG has led to positive results in previous IP-SDM projects, and we hypothesized that adapting its structure to a particular decision by adding relevant information to its content, i.e. location of care options for elderly people, and inviting relevant end-users to steer its adaptation and assess the results, could result in a user-relevant guide that would facilitate the decision-making process.

#### Evidence about the options

The overview of systematic reviews identified 14 eligible systematic reviews. The evidence about impact of care locations on elderly people's health outcomes was highly heterogeneous. Results supported a positive impact of home support interventions on health outcomes. However, there was insufficient evidence to determine the impact of alternative care locations on elderly people's health.

We also obtained preliminary data from an ongoing review update by Mottram et al., which suggests that there is insufficient evidence to draw conclusions about optimal locations of care [26]

#### Drafting first prototype

The following major adaptations were made to the OPDG:

We specified the decision to be made (“To receive the care and services I need, should I stay in my home or move?”).

To encourage unbiased responses, we designated space for elderly people and/or caregivers to write down their own answers, views, and preferences, including that of not making a decision. We added pre-specified answer categories to open questions to facilitate response (e.g., Reasons for making the decision: “I am worried about my health”, “I feel alone”, “I am less able to walk or move around”; Timing of the decision: “As soon as possible”, “Within two to four weeks”, “Within two to six months”).

To determine which options were available based on elderly people’s assessed needs for care and services, we added the functional autonomy profile score (as determined by the Système de Mesure de l’Autonomie Fonctionnelle, or Iso-SMAF [32], used by healthcare professionals in the Province of Quebec) and mapped these to a list of available locations of care options. We described the options in terms of their influence on daily life (setting, space) and support and services available. The guide also encouraged elderly people to obtain more tailored information (e.g., costs, services, support) from their healthcare provider.

As the overview of reviews did not find definitive evidence regarding optimal locations of care for elderly people, we were not able to add evidence-based advantages and disadvantages to the guide, and instead we emphasized the importance of knowing the options available, preferences and values, and (preferred) role in decision making. In addition, we added examples of pros and cons, and general facts about the elderly in the province of Quebec. To help people think about options and preferences, we included in the OPDG “explore your decision” section (also known as a values clarification exercise) a list of examples of pros and cons of staying at home versus moving to another place to get the care and services needed.

We added a question about what else is needed to make the decision (next steps) [20, 29], and added space for questions or comments. We added graphics throughout to aid understanding.

#### First prototype

The first prototype was finalized on September 30, 2014. All relevant minimal IPDAS criteria were fulfilled (Table 1).

### Cycle 2

#### Characteristics of the participants

All five caregivers and two of the five health administrators completed the survey (response rate = 70 %). Median age of the caregivers was 68 years old (range 57–68; *n* = 5) and all were female. Their educational level was college level or higher. Caregivers took care of either one (*n* = 2) or both (*n* = 2) parents, or their husband (*n* = 1). The three health administrators who did not complete the questionnaire provided detailed verbal comments on the guide which addressed many of the survey questions, and were used to improve the guide.

#### Comments and changes (Usability I)

Caregivers and health administrators were positive about the initiative to develop a guide. They thought the guide would be useful for the target population and helpful in decision making (Median = 3 (0–3)). Table 3 provides an overview of the comments and how these were addressed.

**Table 3:** Comments on the first guide prototype (cycle 2)

	Comments	Caregivers	Health administrators	Change
General	Very good initiative	✓	✓	
Length/amount of information	Too long	✓	✓	Shorten text
	Some information is missing, not enough detail about options (for example lists of resources in community)	✓	✓	Remove parts and provide additional document for professionals with relevant information
	Remove list of pros and cons, stimulate people to think for themselves	✓	✓	Remove page, give examples of pros and cons in additional document for HCP
		✓	✓	
Understand ability/clarity of use	Provision of examples	More	Less	We tried to mention examples where possible
	Tool is broad and specific at the same time, and complex to complete		✓	The tool is not meant to be completed alone but with the HCP; this will be emphasized to the HCP when handing out the tool and introducing it
	Consider literacy		✓	Readability checked
	The information about the general data and (lack of) evidence is difficult	✓	✓	Text clarified, general information removed
	Iso-SMAF needs clarification	✓	✓	Text is clarified and shortened, figure updated
Target population	Not consistent to whom is directed/ not clear what target population is (senior, caregiver, professional)	✓	✓	Wording checked, target population clarified in Introduction
	Not clear if the tool is meant to support decision making about moving from any location to any other		✓	Emphasize that the tool is meant for people who live in a traditional home setting and are thinking about moving elsewhere
Balance	(Too) balanced	✓	✓	
	Presentation of costs is leaning a bit towards staying at home		✓	We tried to mention the costs as neutrally as possible
Design/presentation	Bigger font size	✓		Increase font size
	Coloured boxes may be difficult to see	✓		Designer adjusts final design

*HCP* Healthcare professional

#### Length

Caregivers and health administrators were satisfied with the amount of information (Median = 2 (2–3)), but less satisfied with the length of the guide (Median = 2.5 (0–3)), suggesting it was too long. They recommended that some information be removed (e.g. examples of pros and cons, explanation of the Iso-SMAF) and proposed that this information be made available in a supporting document. We could not support the examples with evidence, and it might have created bias. They also suggested adding information about location of care options and costs in the region.

*Subsequent changes* therefore included removing the examples of pros and cons. Instead, people were invited to think of their own pros and cons, consistent with the original OPDG. We also removed part of the Iso-SMAF explanation. Both of these elements were however made available in an additional supporting document to be used if needed, along with information on regional options and resources (e.g., lists of possible residences and types of home-care or community initiatives per region and ways to calculate costs).

#### Understandability

Although health administrators expressed concern that the information was too complicated for elderly people, caregivers experienced no difficulties understanding the content (Median = 2.5 (2–3)) and indicated it was clear how the guide should be used (Median = 2 (1–3)). However, Step 2, or identification of the available options, was considered less clear.

*Subsequent changes* included deleting general information about the elderly in Quebec, rewording the sentence regarding lack of evidence about the better option, and changing the page lay-out to enable better comparison between choices.

We emphasized the importance of elderly people knowing their options, being involved in decision making and clarifying their values with caregivers and healthcare professionals. In the presentation of location of care options we compared available support, services and costs for each option. The information needed to identify available options is to be provided by the healthcare professional in collaboration with elderly people and their families and completed during the consultation session in which options are explored.

#### Lay out

Although quantitative numbers indicate that end-users valued the layout (Median = 2 (1–3), comments indicated that the font size was considered too small and the layout of the guide was poor.

*Subsequent changes* included increasing the font size and spreading out the decision making steps over more pages to make the layout clearer.

#### Second prototype

The second guide prototype, whose final content was confirmed after the usability test, was agreed upon in January 2015. Eight out of 12 IPDAS criteria were fulfilled (Table 1).

### Cycle 3

The prototype that followed usability test adaptations underwent several rounds of review by research team members, caregivers and health administrators. End-users were enthusiastic about the result. For example, one caregiver stated:

*I would like to congratulate you and the whole team, because I am very aware of all the work it [the guide] represents. Good result, and it was a pleasure!*

Caregiver for mother, 68 years old

#### Final guide

**Fig. 1:**
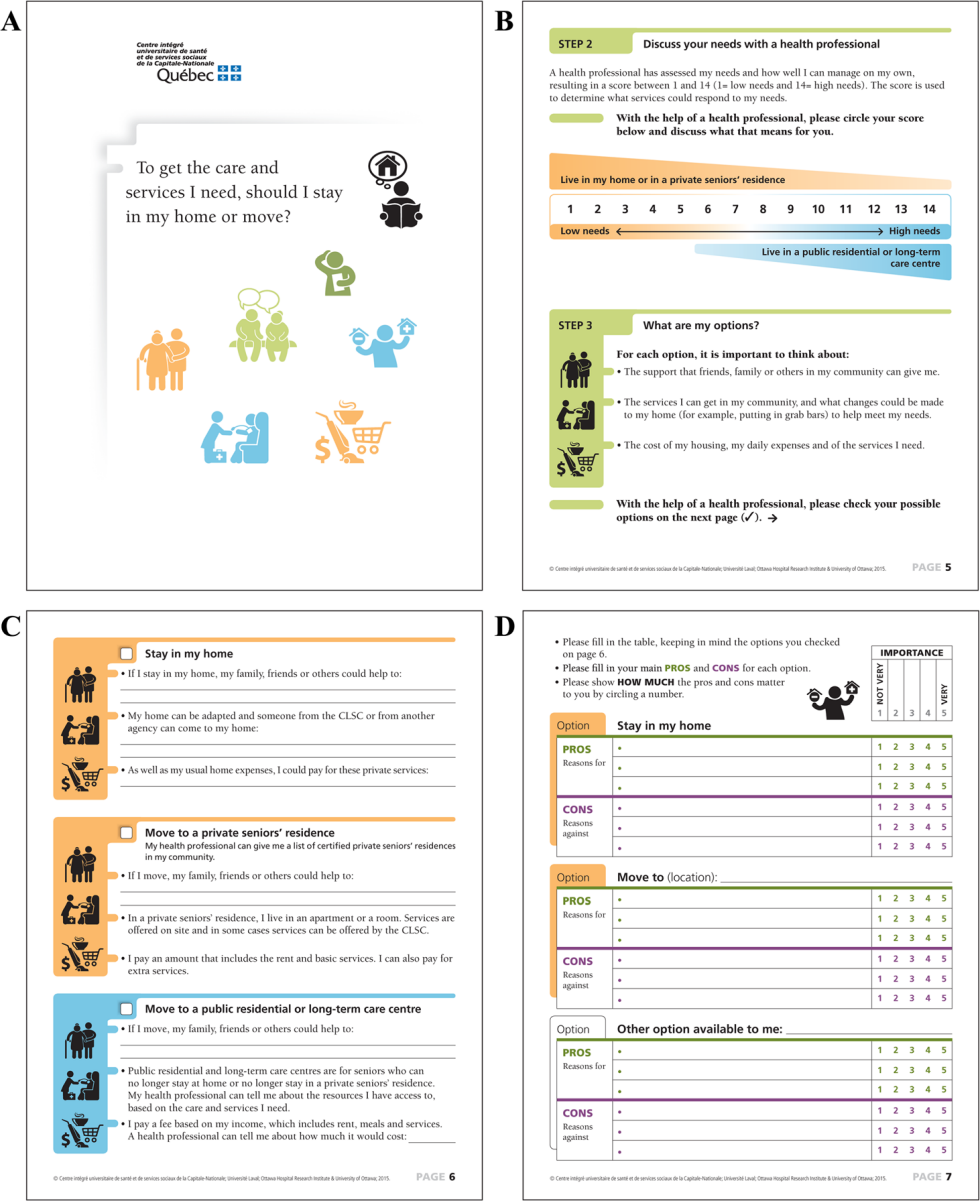
Examples of some pages of the decision guide (English): **a** Front page; **b** Explore your options; **c** Options; **d** Weigh pros and cons

By May 2015, the team had agreed on the final French and English guide, a 10-page coloured booklet printed on A3 sized paper (Fig. 1). It consists of six steps to involve elderly people, caregivers and healthcare professionals in the decision making process about location of care.

#### Readability

The English guide had a readability score of 87.7 (requiring Grade 4.3 level, or four years of schooling) and the score for the French version was 88 (six years of schooling).

#### Usability II

The final guide was assessed for its usability by four elderly people. One was a participant in the DOLCE study, and three were elderly family members of the caregivers involved in the guide development (the others could not be reached (*n* = 2) or were unable to participate due to Alzheimers (*n* = 1)).

The mean age of the elderly people was 82 years; all were female; two were widows, two were married; and their education level ranged from primary school to university graduates. Elderly people thought that the information on all pages was clear (median = 4 (2–4)), the length was just right (4/4), the amount of information was just right (3/4; one thought it was too little), it was balanced (3/4; one thought it was slanted towards moving elsewhere), and that the tool would be helpful for people making the decision about location of care (4/4).

#### Minimal criteria for DAs

Eight out of the 12 qualifying and certifying IPDAS criteria were fulfilled (Table 1). After responding to end-user comments and suggestions, the guide did not describe positive and negative features, nor what it is like to experience the consequences of the options, although this information was provided in a supporting document. We could no longer describe it as a DA and so labelled the guide as a decision guide.

## Discussion

This article reports on the involvement of end-users (elderly, caregivers, and health administrators) in the development of a decision guide for the elderly and their caregivers facing the decision to stay at home or to move elsewhere to receive the care and services they need. Consistent with the OPDG, our guide clarifies the decision to be made, describes each option, and allows listing of pros and cons in a balanced format with an explicit values clarification exercise. The guide was conceived, adapted by caregivers and health administrators, usability-tested and found acceptable by caregivers and elderly people, and found consistent with health administrators’ guidelines and policies. The developmental process leads us to make four main observations.

First, the iterative process that involved end-users facilitated the development of a guide that was found acceptable for our target population. Although DA development is often presented as a linear process [19], the separate development phases are better described as an ongoing cyclical process [14, 32]. An iterative design [14] in which data collection and adaptation are ongoing between each successive prototype is highly applicable to this cyclical process and, moreover, well adapted to user-centered involvement. Due to ongoing feedback, the guide underwent numerous changes. With continuous advances in medicine, changing evidence about the options, and the changing characteristics of end-users, this cyclical process will continue after development of a final prototype [15].

Second, the involvement of end-users was critical to our process, yet it also meant that our original aim of meeting recognized qualifying criteria for DAs was compromised. In response to end-users comments, we removed elements from the integral guide that were necessary to qualify it as a DA according to IPDAS criteria, namely, the overview of examples of pros and cons, and what it is like to experience the consequences of the options. Instead it offered space for writing down pros and cons, and provided the other elements in a separate document. As with other decision support interventions that have failed to meet minimal IPDAS criteria [33], some of the steps in our guide are intended to be filled out with the health professional during a consultation (i.e. with additional verbal information) [33] and fulfillment of the criteria can still therefore be achieved if the decision guide is used along with decision-coaching. In spite of this compromise regarding our final guide, our experience of involving end-users in the development and assessment process at the expense of fulfilling scientific criteria to the letter raises interesting questions about the growing trend of end-user involvement in research, highlighting their cutting-edge role in challenging or finessing scientific models and criteria in the future.

Third, while the involvement of end-users is important, it is also challenging, and indeed the literature confirms that there is much debate about which stage of the research process is most appropriate for end-user involvement [34, 35]. Regarding DA development, there is little evidence about effective methods to involve patients [14, 36]. Moreover, a recent review on patient engagement in DA development found that few DAs are developed with the involvement of vulnerable end-users (M. Dugas et al., submitted 2016). We ensured inclusion of end-users’ expert opinions at every stage of decision guide development, and therewith motivated (vulnerable) end-users for using the decision guide later on. However, end-users occasionally had contradicting opinions about the desired content. Moreover, health administrators were not always able to accurately predict caregivers’ or elderly peoples’ preferences about the content of the decision guide, and vice versa. The inability of clinicians, patients, and caregivers to predict each other’s preferences is also reported in the treatment decision making literature [7, 37–39]. This highlights the importance of involving all possible end-users in the development process and not relying on one stakeholder group to speak for another [14, 40].

The different backgrounds of team members (SDM versus clinical home-care, researchers versus healthcare administrators) also provoked debate about content, which indicated to us that end-users would have different opinions and information needs whatever the guide looked like. Our solution was to summarize the comments of the end-users, noting who made them and weighing the best solution from different (professional) perspectives (e.g., caregivers’ experience with the decision, management and policy, research, practice) and hold the final consensus meetings among team members including the two caregivers, while elderly people evaluated the guide’s usability afterwards. Nevertheless, the final product, whose purpose was to keep the elderly at the very center of the decision making process, excluded the principal end-user at a critical stage. Who should have the final say, in research with interprofessional (healthcare) teams and end-users with different values, perspectives, levels of education, interests and ethical codes [41]? In retrospect, it may have been worthwhile to begin the process by defining a method for weighing everyone’s opinions before embarking on the project. Researchers should develop strategies for working collaboratively with patients, carers and, when appropriate, the public in DA development.

Fourth, the questions asked and suggestions offered by end-users during development clearly indicated the need for clearer explanations of certain steps and options. As the guide will be part of a multifaceted intervention to implement SDM in home-care teams, healthcare providers will be trained in using it to guide elderly people and caregivers and to offer the explanations they need [4]. Other aspects of the intervention include training home-care teams in SDM, including a tutorial, a video about using the guide in an SDM context, and role play. Additionally, in keeping with end-user feedback, the guide comes with a supporting document with detailed resources for the healthcare provider.

One limitation of our study was that although the elderly home-care service users tested the tool’s usability, we did not elicit their feedback in the development process, but their caregivers instead, for reasons explained above. However, the majority of the caregivers (3/5) were over 65 years old and could be considered elderly themselves. They were all women, so we do not know if men would use the guide differently or if its acceptability can be generalized to both sexes. In addition, involving the same caregivers in development and usability testing may have positively influenced the usability test results; a separate independent group of caregivers might have had different views. Future research should assess whether involving the same end-users at the various stages leads to a clinically relevant bias. Future research should also address whether or not the guide is effective for autonomous elderly people, caregiver-senior dyads, or caregivers alone. Secondly, the included caregivers were all somewhat better educated than the general population. This is frequently seen in user-centered research, and is known to be a barrier to the representativeness of members of the public taking part in research teams [42]. Lastly, everyone who was approached participated in our study cycles, but three health administrators provided qualitative feedback instead of completing the survey. Although this limited our quantitative comparison of data about usability of the guide, it provided us with in-depth informative information that aided its further development.

## Conclusion

In conclusion, a user-centered design process was used to develop a paper-based decision guide to support caregivers and elderly in decision making about location of care. The guide was deemed acceptable and understandable to end-users. The impact of the guide is being evaluated in a multicenter cluster randomized controlled trial as part of a larger implementation strategy to increase home-care teams’ practice of SDM with elderly people [4].

## Abbreviations

DA, decision aid; IP, interprofessional; IPDAS, International Patient Decision Aids Standards; Iso-SMAF, l’Instrument Système de Mesure de l’Autonomie Fonctionnelle; OPDG, Ottawa Personal Decision Guide; SDM, shared decision making
